# 1,2-Bis(4-amino­phen­oxy)ethane

**DOI:** 10.1107/S1600536808014736

**Published:** 2008-05-21

**Authors:** M. Saeed Butt, Zareen Akhter, Michael Bolte, Humaira M. Siddiqi

**Affiliations:** a Department of Chemistry, Quaid-I-Azam University, Islamabad 45320, Pakistan; b Institut für Anorganische Chemie, J. W. Goethe-Universität Frankfurt, Max-von-Laue-Strasse 7, 60438 Frankfurt/Main, Germany

## Abstract

The mol­ecule of the title compound, C_14_H_16_N_2_O_2_, is located on a crystallographic twofold rotation axis. The central O—C—C—O bridge adopts a *gauche* conformation. One of the amine H atoms is disordered over two equally occupied positions. The crystal structure is stabilized by N—H⋯O and N—H⋯N hydrogen bonds.

## Related literature

For related literature, see: Barikani & Mehdipour-Ataei (2000 ▶); Eastmond & Paprotny (1999 ▶); Hsio *et al.* (1997 ▶); Liaw & Liaw (2001 ▶); Yang & Chen (1993 ▶); Hergenrother *et al.* (2002 ▶).
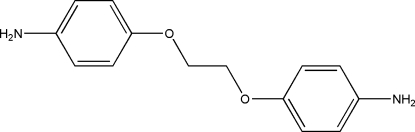


## Experimental

### 

#### Crystal data

C_14_H_16_N_2_O_2_
*M* *_r_* = 244.29
Orthorhombic, 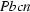

*a* = 14.2157 (9) Å
*b* = 10.4608 (8) Å
*c* = 8.1817 (5) Å
*V* = 1216.68 (14) Å^3^
*Z* = 4
Mo *K*α radiation
μ = 0.09 mm^−1^
*T* = 173 (2) K
0.37 × 0.35 × 0.23 mm

#### Data collection

Stoe IPDSII two-circle diffractometer
Absorption correction: none
15103 measured reflections
1700 independent reflections
1549 reflections with *I* > 2σ(*I*)
*R*_int_ = 0.049

#### Refinement

*R*[*F* ^2^ > 2σ(*F* ^2^)] = 0.048
*wR*(*F* ^2^) = 0.119
*S* = 1.20
1700 reflections
95 parameters
H atoms treated by a mixture of independent and constrained refinement
Δρ_max_ = 0.29 e Å^−3^
Δρ_min_ = −0.19 e Å^−3^

### 

Data collection: *X-AREA* (Stoe & Cie, 2001 ▶); cell refinement: *X-AREA*; data reduction: *X-AREA*; program(s) used to solve structure: *SHELXS97* (Sheldrick, 2008 ▶); program(s) used to refine structure: *SHELXL97* (Sheldrick, 2008 ▶); molecular graphics: *XP* in *SHELXTL-Plus* (Sheldrick, 2008 ▶); software used to prepare material for publication: *PLATON* (Spek, 2003 ▶).

## Supplementary Material

Crystal structure: contains datablocks I, global. DOI: 10.1107/S1600536808014736/zl2110sup1.cif

Structure factors: contains datablocks I. DOI: 10.1107/S1600536808014736/zl2110Isup2.hkl

Additional supplementary materials:  crystallographic information; 3D view; checkCIF report

## Figures and Tables

**Table 1:** Hydrogen-bond geometry (Å, °)

*D*—H⋯*A*	*D*—H	H⋯*A*	*D*⋯*A*	*D*—H⋯*A*
N1—H1*A*⋯O1^i^	0.93 (2)	2.53 (2)	3.4082 (18)	158.6 (18)
N1—H1*B*⋯N1^ii^	0.94 (4)	2.48 (4)	3.360 (3)	157 (4)
N1—H1*C*⋯N1^iii^	0.96 (5)	2.61 (5)	3.468 (3)	148 (3)

Symmetry codes: (i) 
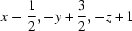
; (ii) 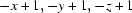; (iii) 
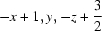
.

**Fig. 1.**
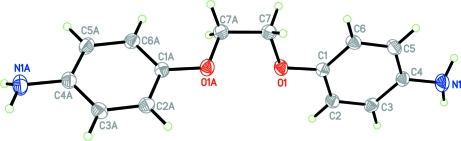
The structure of the title compound showing the atomic numbering scheme. Displacement ellipsoids are drawn at the 50% probability level and only one of the two alternative types of N-H hydrogen atoms is shown. Symmetry code for generating equivalent atoms: (A) -x+2, y, -z+3/2.

## supplementary crystallographic information

### Comment

Aromatic polyimides are well accepted as high performance and heat resistant materials (Hergenrother *et al.*, 2000). They exhibit a favorable balance of physical and chemical properties, show excellent thermal, mechanical and electrical properties and are thus widely used in microelectronics and aerospace engineering (Eastmond & Paprotny, 1999). However, the technological and industrial application of rigid polyimides are limited by processing difficulties due to their high melting or glass transition temperatures and their lack of solubility in most organic solvents (Hsio *et al.*, 1997). Strong interactions between polyimide chains and their rigid structures are the main reason for these behaviors. To overcome such a drawback, different methods have been introduced to modifiy their structures. Many efforts have been made in designing and synthesizing new dianhydrides (Eastmond & Paprotny, 1999) and diamines (Yang & Chen, 1993), and therefore producing a great variety of more soluble and processable polyimides for various purposes and applications. Incorporation of flexible units such as –NHCO–, –O–, (Barikani & Mehdipour-Ataei, 2000), –CO– and –SO2- is one of the most important approaches to overcome these processing problems (Liaw & Liaw, 2001). The title compound is such a new starting material for the synthesis of high performance polyimides.

Molecules of the title compound, C_14_H_16_N_2_O_4_, are located on a crystallographic twofold rotation axis. The central O—C—C—O bridge adopts a *gauche* conformation. One of the amino H atoms is disordered over two equally occupied positions. As a result of that, neighbouring molecules are connected by alternating hydrogen bonds, either N1-H1B···N1^ii^ or N1-H1C···N1^iii^, because H1B and H1C and their symmetry equivalents would be too close to each other and would be mutually exclusive (symmetry codes: see Table 1). In addition, the crystal structure is stabilized by N—H···O hydrogen bonds (Table 1).

### Experimental

A two neck 250 ml round bottom flask was charged with 1 g of 1,2-di(*p*-nitrophenyloxy) ethylene (3.28 mmoles), 10 ml of hydrazine monohydrate, 80 ml of ethanol and 0.06 g of 5% palladium on carbon (Pd/C).The mixture was heated to reflux for 16 h and then filtered to remove Pd/C and the crude solid was recrystallized from ethanol to yield 92.2% of the diamine, m.p. 352K.

### Refinement

All H atoms could be located by difference Fourier synthesis but were ultimately placed in calculated positions using a riding model with C—H(aromatic) = 0.95 Å or *C*—*H*(methylene) = 0.99 Å with fixed individual displacement parameters [U_iso_(H) = 1.2 *U*_eq_(C). The amino H atoms were freely refined. One of the amino H atoms is disordered over two equally occupied positions.

### Crystal data

**Table:**

C_14_H_16_N_2_O_2_	*D*_x_ = 1.334 Mg m^−^^3^
*M**_r_* = 244.29	Melting point: 179 K
Orthorhombic, *P**b**c**n*	Mo *K*α radiation λ = 0.71073 Å
Hall symbol: -P 2n 2ab	Cell parameters from 14232 reflections
*a* = 14.2157 (9) Å	θ = 3.5–29.7º
*b* = 10.4608 (8) Å	µ = 0.09 mm^−^^1^
*c* = 8.1817 (5) Å	*T* = 173 (2) K
*V* = 1216.68 (14) Å^3^	Block, dark red
*Z* = 4	0.37 × 0.35 × 0.23 mm
*F*_000_ = 520	

### Data collection

**Table:**

Stoe IPDSII two-circle diffractometer	1549 reflections with *I* > 2σ(*I*)
Radiation source: fine-focus sealed tube	*R*_int_ = 0.049
Monochromator: graphite	θ_max_ = 29.6º
*T* = 173(2) K	θ_min_ = 3.5º
ω scans	*h* = −19→19
Absorption correction: none	*k* = −14→11
15103 measured reflections	*l* = −11→11
1700 independent reflections	

### Refinement

**Table:**

Refinement on *F*^2^	Hydrogen site location: inferred from neighbouring sites
Least-squares matrix: full	H atoms treated by a mixture of independent and constrained refinement
*R*[*F*^2^ > 2σ(*F*^2^)] = 0.048	*w* = 1/[σ^2^(*F*_o_^2^) + (0.0299*P*)^2^ + 0.7945*P*] where *P* = (*F*_o_^2^ + 2*F*_c_^2^)/3
*wR*(*F*^2^) = 0.119	(Δ/σ)_max_ < 0.001
*S* = 1.20	Δρ_max_ = 0.29 e Å^−^^3^
1700 reflections	Δρ_min_ = −0.19 e Å^−^^3^
95 parameters	Extinction correction: SHELXL97 (Sheldrick, 2008), Fc^*^=kFc[1+0.001xFc^2^λ^3^/sin(2θ)]^-1/4^
Primary atom site location: structure-invariant direct methods	Extinction coefficient: 0.024 (2)
Secondary atom site location: difference Fourier map	

### Special details

**Table:**

Geometry. All e.s.d.'s (except the e.s.d. in the dihedral angle between two l.s. planes) are estimated using the full covariance matrix. The cell e.s.d.'s are taken into account individually in the estimation of e.s.d.'s in distances, angles and torsion angles; correlations between e.s.d.'s in cell parameters are only used when they are defined by crystal symmetry. An approximate (isotropic) treatment of cell e.s.d.'s is used for estimating e.s.d.'s involving l.s. planes.
Refinement. Refinement of *F*^2^ against ALL reflections. The weighted *R*-factor *wR* and goodness of fit *S* are based on *F*^2^, conventional *R*-factors *R* are based on *F*, with *F* set to zero for negative *F*^2^. The threshold expression of *F*^2^ > σ(*F*^2^) is used only for calculating *R*-factors(gt) *etc*. and is not relevant to the choice of reflections for refinement. *R*-factors based on *F*^2^ are statistically about twice as large as those based on *F*, and *R*- factors based on ALL data will be even larger.

### Fractional atomic coordinates and isotropic or equivalent isotropic displacement parameters (Å^2^)

**Table:**

	*x*	*y*	*z*	*U*_iso_*/*U*_eq_	Occ. (<1)
O1	0.91391 (6)	0.60429 (10)	0.65677 (12)	0.0243 (3)	
N1	0.52550 (9)	0.65323 (15)	0.5428 (2)	0.0332 (3)	
H1A	0.5093 (15)	0.718 (2)	0.470 (3)	0.048 (6)*	
H1B	0.496 (3)	0.573 (4)	0.537 (5)	0.042 (11)*	0.50
H1C	0.486 (3)	0.650 (4)	0.638 (6)	0.048 (12)*	0.50
C1	0.81695 (9)	0.61092 (12)	0.63296 (15)	0.0198 (3)	
C2	0.78489 (9)	0.71034 (13)	0.53437 (17)	0.0232 (3)	
H2	0.8285	0.7684	0.4872	0.028*	
C3	0.68899 (10)	0.72479 (13)	0.50483 (17)	0.0239 (3)	
H3	0.6678	0.7931	0.4377	0.029*	
C4	0.62345 (9)	0.64020 (13)	0.57255 (17)	0.0234 (3)	
C5	0.65662 (10)	0.54140 (14)	0.67195 (19)	0.0268 (3)	
H5	0.6131	0.4835	0.7196	0.032*	
C6	0.75275 (10)	0.52624 (13)	0.70255 (17)	0.0240 (3)	
H6	0.7742	0.4586	0.7704	0.029*	
C7	0.94691 (9)	0.49765 (13)	0.75075 (18)	0.0244 (3)	
H7A	0.9230	0.4169	0.7033	0.029*	
H7B	0.9237	0.5043	0.8645	0.029*	

### Atomic displacement parameters (Å^2^)

**Table:**

	*U*^11^	*U*^22^	*U*^33^	*U*^12^	*U*^13^	*U*^23^
O1	0.0192 (4)	0.0272 (5)	0.0264 (5)	−0.0010 (4)	−0.0026 (4)	0.0063 (4)
N1	0.0205 (6)	0.0377 (7)	0.0413 (8)	0.0012 (5)	−0.0023 (5)	0.0043 (6)
C1	0.0202 (6)	0.0214 (6)	0.0178 (5)	−0.0003 (5)	−0.0017 (4)	−0.0017 (5)
C2	0.0231 (6)	0.0230 (6)	0.0235 (6)	−0.0010 (5)	0.0019 (5)	0.0039 (5)
C3	0.0246 (6)	0.0239 (6)	0.0233 (6)	0.0036 (5)	−0.0001 (5)	0.0028 (5)
C4	0.0205 (6)	0.0254 (6)	0.0244 (6)	0.0005 (5)	−0.0010 (5)	−0.0028 (5)
C5	0.0228 (6)	0.0267 (6)	0.0310 (7)	−0.0048 (5)	−0.0004 (5)	0.0043 (6)
C6	0.0247 (6)	0.0223 (6)	0.0250 (6)	−0.0023 (5)	−0.0033 (5)	0.0050 (5)
C7	0.0237 (6)	0.0233 (6)	0.0261 (6)	−0.0007 (5)	−0.0050 (5)	0.0024 (5)

### Geometric parameters (Å, °)

**Table:**

O1—C1	1.3938 (15)	C3—C4	1.3992 (19)
O1—C7	1.4338 (16)	C3—H3	0.9500
N1—C4	1.4202 (18)	C4—C5	1.397 (2)
N1—H1A	0.93 (2)	C5—C6	1.3983 (19)
N1—H1B	0.94 (4)	C5—H5	0.9500
N1—H1C	0.96 (5)	C6—H6	0.9500
C1—C2	1.3928 (18)	C7—C7^i^	1.510 (2)
C1—C6	1.3935 (18)	C7—H7A	0.9900
C2—C3	1.3927 (18)	C7—H7B	0.9900
C2—H2	0.9500		
			
C1—O1—C7	115.93 (10)	C5—C4—C3	118.27 (12)
C4—N1—H1A	115.0 (14)	C5—C4—N1	120.12 (13)
C4—N1—H1B	112 (3)	C3—C4—N1	121.61 (13)
H1A—N1—H1B	120 (3)	C4—C5—C6	121.20 (12)
C4—N1—H1C	115 (3)	C4—C5—H5	119.4
H1A—N1—H1C	114 (3)	C6—C5—H5	119.4
H1B—N1—H1C	75 (3)	C1—C6—C5	119.65 (12)
C2—C1—C6	119.80 (12)	C1—C6—H6	120.2
C2—C1—O1	116.21 (11)	C5—C6—H6	120.2
C6—C1—O1	123.98 (12)	O1—C7—C7^i^	108.83 (10)
C3—C2—C1	120.12 (12)	O1—C7—H7A	109.9
C3—C2—H2	119.9	C7^i^—C7—H7A	109.9
C1—C2—H2	119.9	O1—C7—H7B	109.9
C2—C3—C4	120.96 (12)	C7^i^—C7—H7B	109.9
C2—C3—H3	119.5	H7A—C7—H7B	108.3
C4—C3—H3	119.5		
			
C7—O1—C1—C2	−176.63 (12)	C3—C4—C5—C6	−0.5 (2)
C7—O1—C1—C6	4.11 (19)	N1—C4—C5—C6	179.72 (14)
C6—C1—C2—C3	−0.3 (2)	C2—C1—C6—C5	0.4 (2)
O1—C1—C2—C3	−179.57 (12)	O1—C1—C6—C5	179.62 (13)
C1—C2—C3—C4	−0.2 (2)	C4—C5—C6—C1	0.0 (2)
C2—C3—C4—C5	0.6 (2)	C1—O1—C7—C7^i^	174.05 (12)
C2—C3—C4—N1	−179.62 (14)		

Symmetry codes: (i) −*x*+2, *y*, −*z*+3/2.

### Hydrogen-bond geometry (Å, °)

**Table:**

*D*—H···*A*	*D*—H	H···*A*	*D*···*A*	*D*—H···*A*
N1—H1A···O1^ii^	0.93 (2)	2.53 (2)	3.4082 (18)	158.6 (18)
N1—H1B···N1^iii^	0.94 (4)	2.48 (4)	3.360 (3)	157 (4)
N1—H1C···N1^iv^	0.96 (5)	2.61 (5)	3.468 (3)	148 (3)

Symmetry codes: (ii) *x*−1/2, −*y*+3/2, −*z*+1; (iii) −*x*+1, −*y*+1, −*z*+1; (iv) −*x*+1, *y*, −*z*+3/2.
